# Blockchain-Based Neural Network Model for Agricultural Product Cold Chain Coordination

**DOI:** 10.1155/2022/1760937

**Published:** 2022-05-31

**Authors:** Zhenghao Gao, Dan Li

**Affiliations:** Northeast Agricultural University, Xiangfang District, Harbin City 150000, Heilongjiang Province, China

## Abstract

This paper adopts a blockchain fusion neural network algorithm to conduct an in-depth study on the model of agricultural cold chain coordination. We aim to enable HDFS to meet the demand of storing many small files of various types generated by various stages of agricultural cold chain coordination and then propose an improved balanced merging and index caching strategy based on file types and size grouping. The main three modules are the file preprocessing module, file balanced merging module, and index caching module. The experimental results show that this method can significantly improve the overall performance of HDFS when storing and reading large amounts of small files. Simulation experiments using the UCI test dataset show that the improved spectral clustering algorithm not only reduces the error rate but also significantly reduces the time spent on the clustering process, demonstrating the effectiveness and feasibility of the improved spectral clustering algorithm. The improved spectral clustering algorithm of this paper is used to cluster and analyze nearly one thousand cold chain coordination-related data, and the optimal city is successfully selected as the construction point of a large cold storage transit station. This study can effectively improve the efficiency of cold chain coordination resources and their time utilization and maximize the profit creation for cold chain coordination enterprises, selecting data features for prediction, experimenting with different models and parameters to optimize accuracy, and embedding the resulting learning system for prediction and further operations. The two models of coordination market demand forecasting models and methods are analyzed separately. Finally, after analyzing the prediction results of the two different prediction methods, it is found that it conforms to the actual situation of coordination development in Jiangxi Province. It shows that the coordination market prediction model established in this paper is meaningful and the prediction analysis made has some practical value.

## 1. Introduction

Cold chain coordination has become an important development direction of food coordination by ensuring the quality and safety of food through the low humidity control of the whole process of coordination. With the improvement of living standards, people's demand for green and healthy products is expanding, and perishable products with high quality and freshness requirements, such as fresh food and aquatic products, are gradually gaining recognition, and the cold chain coordination industry is thus ushering in a once-in-a-century development opportunity. Due to the imperfection of domestic cold chain facilities and equipment and core technology management, cold chain transportation costs and energy consumption are high. During the long-distance transportation, in storage and sales of some fresh foods, the state of frozen products is repeatedly frozen and thawed due to the fluctuation of ambient temperature, seriously affecting the quality of frozen products, resulting in food waste and other phenomena [1]. To solve this phenomenon, cloud solutions based on IoT technology are applied in the cold chain, through the collection, transmission, and processing of the actual environmental data in the process of cold chain coordination, to guarantee the safety of cold chain products, realize the value-added products, and achieve efficient management of cold chain operation. The process of cold chain Internet of Things (IoT) therefore generates a huge amount of cold chain inspection information data. In recent years, machine learning algorithms have emerged in various industries, which learn the laws from a large amount of historical data to make intelligent identification of new samples or prediction of specific features to reduce losses and costs [2]. But, in systems such as cold chain sorting, the coverage of intelligent devices using machine learning algorithms is still relatively small, and cold chain operations still consume a lot of human and material resources. How to combine the massive cold chain inspection information data with machine learning algorithms for processing, analysis, and application, to obtain useful information value and improve the operation process, becomes a question worth thinking about.

The cold chain coordination of agricultural products needs to connect the scattered demand and supply sources of agricultural products. Since there are many participants in the supply and demand of agricultural products, and the number of information exchanges is also large, a huge amount of data will be generated in the whole process of agricultural cold chain coordination [3]. To effectively maintain the information exchange between the supply and demand sides of agricultural cold chain coordination and effectively manage these information data, it is necessary to process these information data with the help of big data technology, to improve the efficiency of agricultural cold chain coordination. Although the development of China's cold chain coordination has been very rapid in the past few years, and the technology related to big data has been relatively mature, there are still more problems in the management of agricultural cold chain coordination based on big data, for example, the lack of a deep and correct understanding of the whole cold chain state of low temperature, the lack of precooling steps for agricultural products, resulting in a significant shortening of the shelf-life of agricultural products, intermittent closure of refrigeration equipment, and transit stations [4]. The size of the node's trust value is between 90 and 100. After the node competes for the accounting right and successfully completes the accounting, the coin age of the node is cleared. The value of *ω* is 0.4, and the node is rewarded with 0.4 trust value; when the node's trust value is 0.4, the size of the node's is between 80 and 90. The interruption of the cold chain due to improper operation during loading and unloading of goods happens from time to time, and the quality of agricultural products is damaged by not maintaining the whole process of refrigeration. Therefore, it is necessary to detect and obtain relevant data in the cold chain coordination of agricultural products in real-time and to be able to divide the data reasonably and analyze them in time to respond appropriately.

The research objectives of this paper are twofold: first, to process cold chain coordination detection data to provide high-quality data for machine learning-based training models. The second is to build a pricing model for cold chain inspection data to price the cold chain coordination inspection data for trading and to exploit the commercial value of the data. In this paper, a new feature selection method is established to process the cold chain coordination inspection data to train the shelf-life prediction model to get the desired results, which can support the intelligent decision-making of the cold chain. In addition, based on the property that Shapley value can be fairly assigned to cooperative alliances, it is applied to the cold chain fishery product intelligent sorting prediction algorithm, which can assign the contribution value of input data information features and provide interpretable data results to measure the prediction cost and prediction accuracy in the intelligent algorithm and provide theoretical support for the cold chain intelligent sorting prediction system. The cold chain coordination management system of agricultural products based on big data is a complex system engineering; in particular the coordination distribution tasks present the complex status of multiple batches and small lots, and the various aspects of the cold chain coordination of agricultural products will generate a huge amount of data that need to be stored and processed, and the storage of a large number of small files seriously affects the computer performance, so it is necessary to find a suitable small file merging model. The cold chain coordination has strict requirements for different agricultural products in terms of temperature and humidity, oxygen content, transportation time control, etc. The modeling needs to consider many constraints and is more complicated.

## 2. Related Works

From the whole process of agricultural products cold chain coordination, the refrigeration environment is the key to the whole cold chain. The current quantity and quality of cold storage, as well as refrigerated trucks, cannot meet the needs of the rapid development of cold chain coordination. Compared with ordinary warehouses, the construction period of cold storage is long, the investment is large, and the versatility is low; the general characteristics of refrigerated transport trucks are also different from those of ordinary trucks, which require a longer manufacturing cycle and higher price [5]. The construction of modern agricultural products cold chain coordination system can not only improve the operational efficiency of the agricultural products industry chain but also meet consumers' demand for green and healthy consumption. From the perspective of macro development of the government, government departments should provide reasonable and necessary resource support for agricultural products cold chain coordination enterprises and play the role of top-level design. The construction of a cold chain coordination system will certainly raise the transportation cost of related enterprises and increase their economic burden [6]. Therefore, consumers should also establish the concept of quality and promote the development of the cold chain coordination industry of agricultural products through the improvement of market demand. In addition, consumers should consider the health of their family members and choose fresher agricultural products delivered by cold chain coordination, thus encouraging enterprises to carry out cold chain coordination delivery [7].

A BP-ANN model was developed to be able to predict the IMP loss of silver carp during 0–3 days of heat treatment. The discriminant analysis model established by BP neural network was studied to establish the discriminant analysis model of the original sea area by the content of heavy metals in the aquatic product samples to realize the traceability of the origin of aquatic products. Based on the comparative analysis of the sensory evaluation, conductivity, and K value of rainbow trout meat at different storage temperatures with storage time using two models, Arrhenius and ANN, the ANN model was shown to be more accurate in predicting the shelf-life of rainbow trout [8]. A BP neural network prediction model was developed to predict the quality and shelf-life of eggs at different temperature ranges. Using the theoretical basis and generalization capability of physical heat transfer models, a flexible neural network framework that can predict the temperature of perishable foods in real-time was developed, capable of estimating the temperature at locations where measurements were not made using temperatures measured by a limited number of sensors placed at critical locations in the shipment [9].

ZigBee and WSN wireless sensor networks are applied to the cold chain coordination real-time monitoring system to achieve multipoint data collection in cold chain carriages [10]. For the real-time function of synchronous temperature monitoring, the application of RFID technology to outlier detection is discussed. Intelligent decision support for cold chain coordination based on machine learning is reflected in all aspects of the cold chain coordination business process [11]. The higher the probability of the node competing for the accounting right, the more times the node has malicious behavior, and the lower the probability of the node competing for the accounting right. For the cold chain coordination transportation process, machine learning-based monitoring and early warning can be carried out to guarantee the quality of coordination products in real-time; for the real-time planning of vehicle distribution paths, intelligent scheduling can be carried out through machine learning methods; meanwhile, cold chain coordination IoT can be used for shelf-life prediction of perishable foods, aquatic products, etc. Temperature is crucial to the quality and safety of fresh agricultural products in the cold chain, and for perishable products, real-time temperature information can be translated into a prediction of their remaining shelf-life, which can then be used to improve supply chain management [12]. In recent years, artificial neural networks have been gradually applied in the shelf-life prediction of aquatic products [13]. The advantage of artificial neural networks is that they can synthesize multiple indicators without considering the relationship between parameters to reduce systematic errors, and they improve the accuracy of prediction through continuous learning. Therefore, scholars have studied many effective methods combined with artificial neural network.

## 3. Blockchain Fusion Neural Network for Building Analysis of Agricultural Cold Chain Coordination

### 3.1. Blockchain Fusion Neural Network Algorithm Design

In the public chain, all the nodes have an equal relationship with each other, and the nodes all adopt the same consensus mechanism, and the data are stored in each node in a distributed manner to ensure the consistency of data throughout the blockchain. In the blockchain network, there is no need to set up any centralized management body, and each node is not dominated by other nodes, and the loss of some nodes has almost no effect on the data integrity of the blockchain system, so the impact resistance of the whole blockchain system is higher compared with the traditional centralized system. Blockchain adopts a consensus mechanism, which is no longer decided by a single organization or institution [14]. The growth of blockchain is through arithmetic to compete for bookkeeping rights, and each node can participate in the competition to jointly maintain the integrity and legitimacy of the whole blockchain, and the data cannot be tampered with and is not interfered with by human factors, so users can conduct transactions and other operations in this system without centralized management mechanism. At the same time, the storage method of the blockchain can handle user data confidentially, and it can enable users to trace any transaction on the blockchain as well as verify the legitimacy of the information. Developers can design their applications based on this blockchain according to their needs, and the whole blockchain system remains transparent and open. The storage structure is like a data structure in the form of a chain table. The next block header contains the hash value of the previous block, which is used to link two adjacent blocks, and once the block is confirmed, it cannot be tampered with or deleted, and the corresponding hash value will be changed if it is maliciously altered, so the blockchain has good security in data protection.

Asymmetric encryption algorithm generates two different keys, i.e., private key and public key. The public key can be provided to all users, while the private key cannot be disclosed and can only be held by oneself. The two secret keys exist in pairs, and the two are jointly involved in encrypting and decrypting the information. The asymmetric cryptographic algorithm can be used to verify the correctness and authenticity of each other's information and identity when interacting with information on the network so that the information can be transmitted securely and stealthily. Data encrypted with the public key of a pair of secret keys must be decrypted with the private key of the pair of secret keys [15]. The digest of the message, like the fingerprint of a person, can uniquely identify the message, and then the digest is encrypted with the private key of the message source, and finally, a digital signature is obtained. The digital signature and the message will be passed to the receiver together, and after receiving it, the receiver will decrypt the digital signature with the public key of the message source, get the message digest encrypted by the previous message source, and compare it with the digest calculated by the message that is received to confirm that the message is correct.

The univariate linear regression model reflects the relationship between one independent variable and the dependent variable, while the multiple linear regression model reflects the relationship between multiple independent variables and the dependent variable, and the principles between them are similar. Multiple regression is an extension of one-variable regression, except that it is relatively complex in terms of calculation. The model of multiple regression can be expressed as

(1) $$\begin{array}{c} Y=\alpha_{0}+\alpha_{1}X_{1}+\alpha_{2}X_{2}+\ldots +\alpha_{m}X_{m}+\beta . \end{array}$$

The layers of a neural network interact and connect with each neuron on nonidentical layers to form a complete system, which is adaptive, self-learning, and capable of processing information. The connection weights and thresholds are in the process of dynamic change, while the accurate prediction values are continuously output.

**Figure 1:**
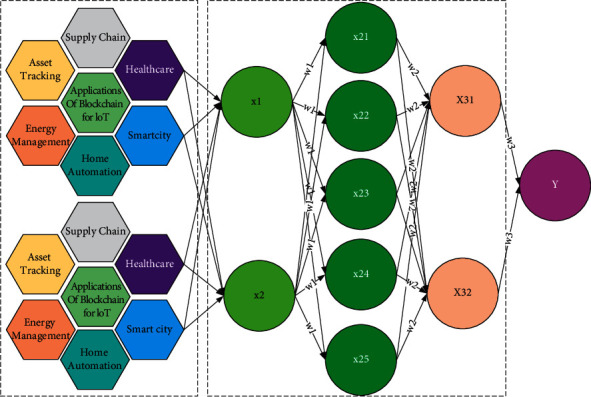
Structure of BP blockchain fusion neural network.

The BP neural network structure consists of an input layer, an intermediate layer, and an output layer, where the information is propagated forward while the errors are propagated backward. The layers can be interconnected with each other and the degree of information received is determined by the network connection weights. Each layer consists of a certain number of neurons, and the neurons in the same layer cannot be connected. One of the intermediate layers can in turn contain multiple layers, while the input and output layers generally have only one, but there can be more than one. It is difficult to fully fit individual outliers. From a practical point of view, the possible reason is that behind any economic phenomenon is the result of the comprehensive movement of various social factors. The neurons in the input layer are responsible for receiving signals from the external environment and then passing them to the intermediate layer. The intermediate layer processes and transforms the signals and can be designed with multiple layers if needed. The intermediate layer processes the received signals, and the processed signals are passed to the output layer, which is responsible for outputting the results to the external environment, thus completing the positive propagation of information [16]. When the predicted value provided by the output layer is not consistent with the expected result, a prediction error is formed. Immediately afterward, the backward propagation process of the error begins, and the error is gradually passed from the output layer to the output layer, while the connection weights of each layer are updated with the gradient descent method. The process of continuously correcting the connection weights in the input and intermediate layers, as well as the output layer, is a continuous learning process of the neural network and a continuous forward and backward propagation of information. The structure of the BP neural network is shown in Figure 1.

The error signal is backpropagated by the network, and the network formed in this way has a strong nonlinear mapping capability. Error backpropagation means that the difference between the output value and the actual desired result is used as the error, and the connection weights of each layer from the output layer to the input layer are gradually adjusted to obtain the minimum error. Usually, the application of neural networks with one intermediate layer is more common. Usually, there is a certain error between the predicted and actual values of the neural network output, and the general expression of the error *E*_*k*_ is

(2) $$\begin{array}{c} E_{k}=\frac{1}{4}\sum_{i=}^{m}{\left(d_{i}+y_{i}\right)}^{3}. \end{array}$$

The neural network uses the desired output value as a reference for the learning process and continuously approximates this value through learning. When the neural network receives an input signal from the outside world, it will communicate the signal to the output layer in a forward propagation manner, and if the actual output value of the output layer does not meet the expectation, the error signal will be backpropagated to update the training parameters.

(3) $$\begin{array}{c} E_{k}=\frac{1}{4}\sum_{g=1}^{h}{\left[d_{g}+f\left({\text{net}}_{g}\right)\right]}^{2}. \end{array}$$

Further expansion to the input layer has

(4) $$\begin{array}{c} E_{k}=\frac{1}{4}\sum_{g=1}^{h}{\left\{d_{g}+f\left[\sum_{j=0}^{m}w_{jg}f{\left({\text{net}}_{j}\right)}^{2}\right]\right\}}^{2}. \end{array}$$

**Figure 2:**
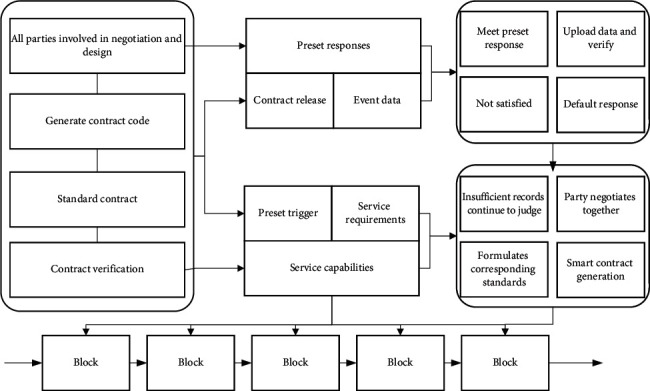
Smart contract operation mechanism diagram.

Smart contracts are the key to the decentralization of blockchain, which makes the whole execution process change from the active execution of uncertainty to the passive execution without the maintenance of third parties, ensuring irreversible and tamper-proof execution, and extends the distributed applications for blockchain, which can provide a more secure and efficient storage environment for the storage of agricultural traceability data based on blockchain. The life cycle of a smart contract includes three stages, contract generation, release, and execution, and its operation mechanism is shown in Figure 2.

In the process of smart contract generation, each participating party negotiates together, clarifies its authority and obligations, formulates corresponding standards, and verifies them to ensure their accuracy. Each node will check and verify the received hash value with its calculated value several times to reach consensus and finally broadcast to each node utilizing a block through the consensus contract collection; after triggering the preset conditions in the contract, the contract will be executed automatically and the result of the contract execution will be sent to each node by the consensus contract collection [17]. After triggering the predefined conditions in the contract, the contract will be executed automatically, and the execution result of the contract will be changed automatically by the consensus of each node, and finally, the whole process will be processed automatically by the smart contract at the bottom of the blockchain. The same agricultural product traceability data is stored, and after 1000 rounds of consensus, 10 experiments are carried out on the number of times each node competes for accounting, and the average number of times each node competes for accounting rights in the results of the 10 experiments is taken.

(5) $$\begin{array}{c} R=a*G=\left(r^{2},r^{\prime }\right),\\ S=a^{2}\left(H\left(m\right)-k*r\right)\text{mod}p. \end{array}$$

In the blockchain, each block is a carrier for storing data summary information, and each block is connected according to its generation time order, forming a chain storage structure. The chain storage structure is the most basic storage structure of all blockchains. The first created block is called the creative block, and the height of the block is 0. After that, the height of the block is increased by 1 for each additional block, and the hash value of the previous block is recorded in the block header. Each block in the blockchain, except the first created Genesis block, is connected by connecting the hash value of the block header in the previous block, and each transaction data stored on the chain will be hashed through layers to get the value of the Merkle tree root stored in the block header, and the hash value of the block header will become completely different with the change of the Merkle tree root value.

(6) $$\begin{array}{c} \left(r_{x}^{\prime },r_{y}^{\prime }\right)=S^{-1}\left(H\left(m\right)-k*r\right)r_{x}^{\prime }\text{mod}p+r_{y}^{\prime }K. \end{array}$$

Due to the high security of the elliptic curve digital signature algorithm, the computational relationship between public and private keys is one-way, only the private key *k* can be known to compute the public key *K*, and the reverse computation is not possible. Therefore, if any slight changes occur in the data during the storage process, the obtained summary data are not the same. In the whole storage process of agricultural traceability data, to reduce the storage pressure of the blockchain, hash calculation is performed on the agricultural traceability data to obtain the data summary information and then stored on the chain to reduce the space for data storage on the chain. Meanwhile, the local database is used to store the corresponding detailed agricultural product traceability data, and ECDSA digital signature algorithm is adopted to verify the security of agricultural product traceability data.

### 3.2. Analysis of the Construction of the Agricultural Product Cold Chain Coordination Model

**Figure 3:**
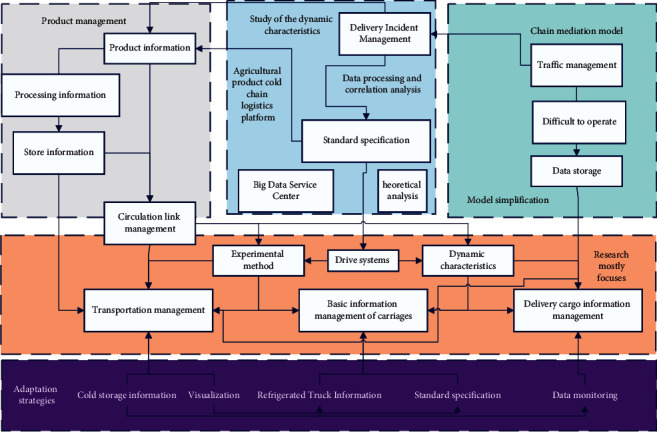
System function relationship diagram.

The management system of agricultural product cold chain coordination based on big data is designed to optimize the management process of each link of agricultural product cold chain coordination, and the management aspects involved can be divided into a big data center and four management links, as shown in Figure 3. The four management links are product management, market supply, and demand management, storage management, and transportation management.

The big data dispatching center includes data storage, data monitoring, data processing, correlation analysis, information visualization, and other functions, which can meet the whole process of safe and effective storage, aggregation, processing, and service dispatching of the massive data of each link of agricultural products cold chain coordination, thus helping the relevant management personnel to understand the status of agricultural products, market supply, and demand, warehouse status, and vehicle transportation status in time and make scientific and objective decisions in time [18].

After cleaning and processing the information of agricultural products, market supply, demand orders, warehouses, and vehicle transportation, different types of data will be categorized for storage management, and each category of data will be stored and counted in a distributed manner. Reducing the probability of nodes doing evil plays a positive role in the efficiency of each node participating in the consensus in the blockchain network and improving the fairness and stability of each node participating in the consensus in the blockchain network. At the same time, the cold chain coordination management system will generate many small files in each link, and Hadoop storage of many small files will lead to a significant decline in storage and computing performance, based on this paper proposing an improved balanced merging and index caching strategy based on file type and size grouping.

Different types of files such as Mysql, Hive, and HBase can be found, deleted, newly created, and updated, and the integrated relevant data information can be mined in the next step, and the mined information can be converted into services to meet various needs of users. It is concluded that different clustering algorithms have their blind spots and misconceptions for different data computation processes, while an improved adaptive spectral clustering algorithm (DCSC-NJW) based on local standard deviation and optimized initial center is proposed for cold chain coordination data analysis based on several characteristics of cold chain coordination data and the spatial distribution characteristics of cold chain coordination data.

Machine learning and artificial intelligence technologies, as advanced automated processes, are extremely dependent on large, real-time data and information flows [19]. Cold chain IoT technology can provide the inspection data information needed to train the model for a machine learning-based fish shelf-life prediction model. Processing the collected data using machine learning algorithms generally involves identifying the learning task, collecting, and cleaning the training data, selecting data features for prediction, experimenting with different models and parameters to optimize accuracy, and embedding the resulting learning system for prediction and further manipulation.

Experiments are conducted here using a set of publicly available datasets from the UCI Machine Learning Repository to verify the model validity. With the improvement of living standards, people's demand for green and healthy products is expanding, and perishable products such as fresh and aquatic products that require high quality and freshness are gradually being recognized. This experimental dataset is used to predict the acute toxicity of a set of 908 chemicals to black-headed dull fish. Acute toxicity refers to the effect of poisoning or even death caused by humans or animals after a single exposure to exogenous chemicals and can indicate the speed and severity of poisoning caused by exogenous chemicals in humans or animals, and the speed and severity of poisoning often lead to different results depending on the quality and quantity of chemicals to which the test animals are exposed.

**Figure 4:**
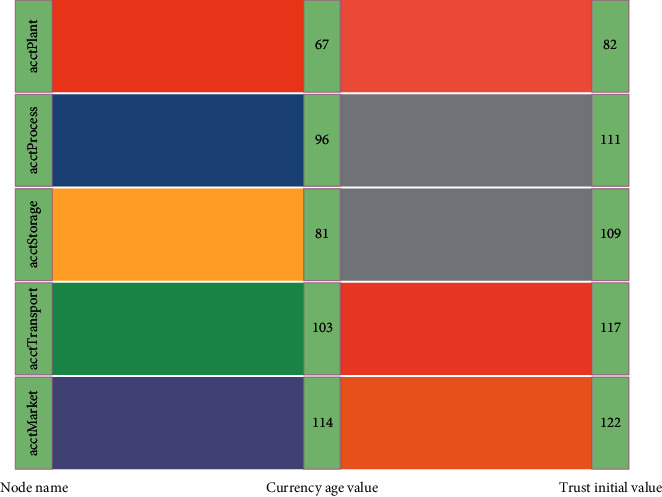
The initial value of trust for which each node corresponds.

At the same time, if there is a node in the blockchain network with a small number of evils, the trust value of the node is still within the range of competing for bookkeeping rights after the node has been punished by the trust for malicious behavior, and the difficulty of finding random numbers when the node is competing for bookkeeping rights is limited by the small trust value. The more the nodes do evil, the more trust penalties they receive, the lower the trust value will be, and the more difficult it will be for nodes to find random numbers when competing for bookkeeping rights in the consensus process, as shown in Figure 4.

When the node has malicious behavior and the trust value is deducted, the size of the node trust value is between 90 and 100, and the node competes for the bookkeeping right and completes the bookkeeping successfully, the node's coinage is cleared, *ω* takes the value of 0.4, and the node is awarded 0.4 trust value; when the node's trust value is between 80 and 90, the node competes for the bookkeeping right and completes the bookkeeping, the node's coinage is cleared according to the rule [20]. To ensure the safety of cold chain products, realize product value-added, and realize efficient management of cold chain operation, “smart logistics” has gradually become a new development trend, and a large amount of cold chain detection information data is generated in the process of cold chain Internet of Things. When the node's trust value is between 70 and 80, the node competes for the bookkeeping right and completes the bookkeeping, the node's coinage is cleared, and *ω* takes the value of 0.2, but the node will be rewarded with 0.2 trust value; when the node has multiple malicious behaviors, the node's trust value is deducted several times, and the node's trust value is between 60 and 70, the node competes for the bookkeeping right and completes the bookkeeping successfully. When the node's trust value is between 60 and 70 and the node competes for the bookkeeping right and completes the bookkeeping successfully, the node's coinage is cleared and *ω* takes the value of 0.1, giving the node 0.1 trust value bonus.

## 4. Results Analysis

### 4.1. Algorithm Performance Results

**Figure 5:**
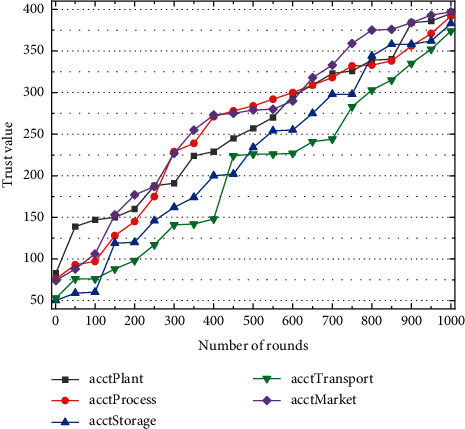
Growth of node trust value based on CPOs consensus mechanism.

Since the node act plant is the evil node that is restricted from participating in node bookkeeping, the probability of the node act plant competing for the bookkeeping right is 0. Node comarket has the highest trust value for the node with a high coinage value, so the probability of this node competing for the bookkeeping right is the highest. Although the trust values of the nodes act process, factorage, and cotransport are relatively low, they are still within the range of trust values that can compete for bookkeeping rights, and as the normal behavior of these nodes accumulates, the trust values gradually increase and the number of times that the nodes compete for bookkeeping rights increases. Therefore, the probability of a node competing for bookkeeping rights is proportional to the trust value of the node: the more honest the node behaves, the higher the trust value of the node is and the higher the probability of the node competing for bookkeeping rights is; the more times the node behaves maliciously, the lower the probability of the node competing for bookkeeping rights is. Even with the nodes that have had malicious behaviors but the trust value is still within the range that they can compete for bookkeeping rights, they will get more bookkeeping rights throughout the consensus process because of their normal behavior competition and trust accumulation, which has a positive impact for each node to compete for bookkeeping rights normally, as shown in Figure 5.

Due to the trust reward and punishment allocation system set in the trust-based CPOs consensus mechanism, the size of each node's trust value in the blockchain network is affected by the behavior of its nodes, and the more the nodes do evil, the lower the degree of being trusted is, and the greater the trust punishment is that nodes receive in the process of participating in consensus. Therefore, a large amount of data will be generated in the whole process of the circulation of agricultural cold chain coordination. To effectively maintain the information exchange between the supply and demand ends of agricultural cold chain coordination and effectively manage these information data, it is necessary to process these information data with the help of big data technology, to improve the efficiency of agricultural cold chain coordination. The difficulty of finding random numbers when each node competes for bookkeeping rights changes continuously with the node's trust accumulation, and the higher the trust value, the lower the difficulty of finding random numbers when competing for bookkeeping rights, and thus the higher the probability of being able to compete for bookkeeping.

**Figure 6:**
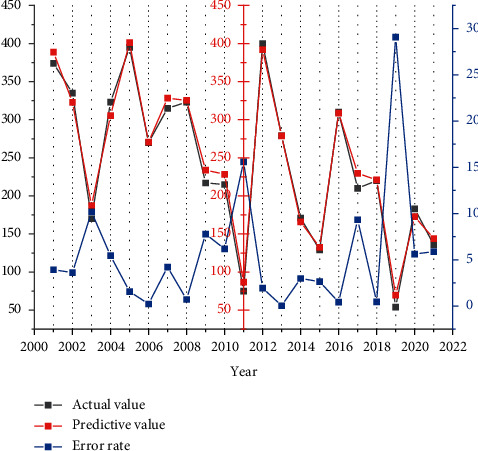
Prediction results and error rate.

The lower the trust value is, the more difficult it is for nodes to find random numbers in the competition for bookkeeping rights, the slower the trust value grows, and the lower the probability of being able to compete for bookkeeping rights is, so the node with the highest node trust value, comarket, has the most significant trust value growth during the 1000 rounds of consensus on traceability data storage, but these nodes have the most significant trust value growth as the amount of data storage increases and the number of rounds each node participates in consensus increases. However, the trust value growth of these nodes stabilizes as the data storage volume increases and the number of rounds each node participates in the consensus increases. The other three nodes, act process, factorage, and cotransport, are limited by the corresponding competition for bookkeeping rights in the process of participating in consensus, but with the active competition of their nodes, the trust value also keeps increasing, which can largely avoid the unfair phenomenon of trust value monopoly when the nodes participate in consensus due to the excessive growth of the nodes' trust value. The unfair phenomenon of nodes' trust value monopoly when participating in consensus is largely avoided as shown in Figure 6.

Except for the prediction results of 2000–2022, the prediction errors of other years are within the acceptable range. For the years with large forecast errors, the possible reason for the analysis from the mathematical perspective is that the model is a linear model, which is difficult to fit completely for individual outliers. To measure the prediction cost and prediction accuracy in the intelligent algorithm, it can provide theoretical support for the cold chain intelligent sorting prediction system. The possible reason for the analysis from the realistic perspective is that behind any economic phenomenon is the result of the integrated movement of various social factors, and the change of freight turnover in different years may be caused by a variety of reasons, and the value-added of the secondary industry is only one factor to measure the change. Therefore, when applying the model to social reality, it cannot be analyzed from a quantitative perspective alone, but should also consider nonquantitative factors such as government policies, industry regulations, and macroenvironment.

### 4.2. Experimental Results of the Agricultural Product Cold Chain Coordination Model

All distribution vehicles start from a single distribution center and return to the original distribution center after serving all customers on the specified route, which is the single distribution center vehicle path problem, which is generally applicable to small-scale intracity distribution. Several distribution centers assign tasks to distribution vehicles of each center at the same time, and after each distribution vehicle has served all customers on the specified route, it returns to its respective starting point, which is the multidistribution center vehicle path problem, which is generally applicable to the situation of larger scale or multiple cities with customer demands.

**Figure 7:**
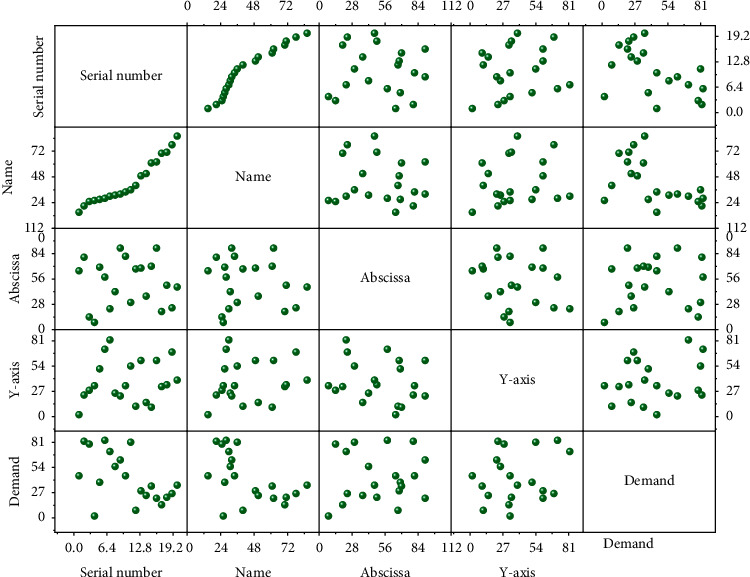
Distribution center information.

The vehicle path problem can be divided into closed vehicle path problem and open vehicle path problem according to whether the distribution vehicles return to the original distribution center point or not. Open vehicle path problem has multiple distribution centers; in general, the distribution vehicle starts from a distribution center, and after completing the designated task, it does not need to return to the original distribution center but adopts the principle of proximity and stays at the distribution center nearest to the end customer point of the vehicle, so that the return distance can be reduced and the total distribution cost can be minimized. Each node can participate in the competition to jointly maintain the integrity and legitimacy of the entire blockchain, and the data cannot be tampered with or interfered with by human factors. Users can conduct transactions and other operations in this system without a centralized management mechanism. A closed vehicle path problem means that the distribution vehicle starts from the distribution center and returns to the starting point after completing the specified task. This mode may be relatively costly, but it is easy to manage the distribution vehicle and it is suitable for distribution problems in a small area, as shown in Figure 7.

The distance between the distribution center and the customer node and between the customer node and the customer node is the linear distance between them. The two consensus mechanisms, PoS consensus mechanism and trust-based CPOs consensus mechanism, proposed in this paper store the same agricultural traceability data with the same node coinage value and conduct 10 experiments on the number of times each node competes to bookkeeping after 1000 rounds of consensus and take the average of the number of times each node competes to bookkeeping right in the 10 experimental results.

**Figure 8:**
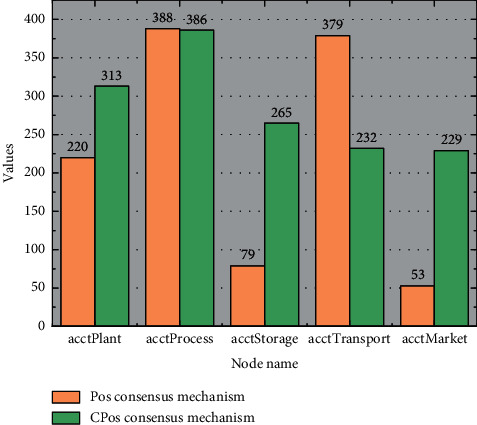
Statistics of the number of bookkeeping rights for the two consensus mechanisms.

Using the PoS consensus mechanism, the number of times each node competes for bookkeeping rights is proportional to the size of each node's coinage, and the larger the node's coinage is, the more times it competes for bookkeeping rights. The node act plant has the lowest coinage value and competes for the least number of bookkeeping rights in the PoS consensus mechanism, but because of the node's high trust value, the number of times the node competes for bookkeeping rights increases when using the CPOs consensus mechanism; and even if the node act process has a high coinage value, because of the node's low trust value, the consensus process may be over. The middle layer processes and transforms the signal, and if necessary, the middle layer can be designed with multiple layers. The intermediate layer processes the received signals, and the processed signals are transmitted to the output layer, which is responsible for outputting the results to the external environment, thus completing the forward propagation of information. The number of times this node competes for bookkeeping rights in the CPOs consensus mechanism decreases compared to the number of times it competes for bookkeeping rights in the PoS consensus mechanism; although node factorage has a relatively small coinage value and competes for bookkeeping rights in the PoS consensus mechanism less often, the trust value of this node is high, so the number of times it competes for bookkeeping rights based on the positive performance of this node significantly increases, which improves the fairness of node competition to a large extent, as shown in Figure 8.

When the number of transactions is small, the delay time of the nodes to issue blocks is comparable to that of the PoS consensus mechanism based on competition in the literature, but as the number of transactions increases, the CPU consensus mechanism proposed in this paper, influenced by the trust reward and punishment mechanism, promotes each node in the blockchain network to actively participate in the competition and continuously accumulate trust values, which reduces the probability of malicious nodes appearing in the blockchain network, improves the efficiency of nodes out of blocks, and shows a significant advantage in delay time. The smart contract used in the process of realizing the traceability data storage of agricultural products based on blockchain technology is that the participants in each link of the agricultural product supply chain formulate corresponding contract standards according to their own authority; the contract needs to be signed by all participants during the release process, and then it is sent to the whole network through the peer-to-peer network and the hash value of the contract received during this period is calculated. Therefore, the trust-based CPOs consensus mechanism designed in this paper not only reduces the probability of malicious nodes appearing but also plays a positive role in the efficiency of each node participating in consensus in the blockchain network.

Finally, we analyze the performance of the blockchain-based traceability data storage scheme and the trust-based CPOs consensus mechanism proposed in this paper from three aspects of security, dynamism, and time delay, respectively, and conclude that the blockchain-based agricultural traceability data storage scheme designed in this paper can ensure the secure storage of traceability data while alleviating the storage pressure of the block. Meanwhile, the trust-based CPO consensus mechanism proposed in this paper can dynamically manage the behavior of nodes based on their trust values, reduce the probability of nodes doing evil, play a positive role in the efficiency of each node participating in consensus in the blockchain network, and improve the fairness and stability of each node participating in consensus in the blockchain network.

## 5. Conclusion

Automated process operations based on technologies such as machine learning and artificial intelligence are beginning to cause changes in the coordination industry due to their ability to complete the required transportation tasks in the cold chain quickly, accurately, and with quality and quantity. However, data-driven automated processes rely on the high quality of data. Both decision processing support for cold chain coordination and management applications rely on the processing and analysis of inspection data obtained from, for example, IoT inspection systems. This paper investigates the problem of feature selection for cold chain aquatic products' shelf-life prediction based on machine learning. Detailed requirement analysis of the supply chain trust system is done, and the environment required for the whole system is installed and deployed, including the underlying blockchain network and Ether wallet, etc. Then key functions such as the protection of key privacy information of enterprises and interenterprise transaction behavior are realized through the writing of smart contracts, and the design of the implementation interface is carried out. A random forest-based shelf-life prediction model for cold chain aquatic products is defined. The agricultural product traceability data is hashed to obtain the data summary information and then stored on the chain to reduce the storage space of the data on the chain. At the same time, the local database is used to store the corresponding detailed agricultural product traceability data, and the ECDSA digital signature algorithm is used to verify the security of the agricultural product traceability data. For the feature selection problem of the prediction process, the recursive feature elimination idea based on cross-validation is combined with feature permutation and combination, and the prediction accuracy is considered together to design a feature selection method combining the two. Compared with the CV-RFE algorithm, the proposed method not only considers the feature selection problem based on the incremental prediction accuracy but also adapts to the feature selection under different prediction costs, to select the most valuable input features for the cold chain product shelf-life prediction model and reduce the loss during transportation.

## Data Availability

The data used to support the findings of this study are available from the corresponding author upon request.
